# Regulatory T Cells in the Pathogenesis and Healing of Chronic Human Dermal Leishmaniasis Caused by *Leishmania (Viannia)* Species

**DOI:** 10.1371/journal.pntd.0001627

**Published:** 2012-04-24

**Authors:** Daniel Rodriguez-Pinto, Adriana Navas, Víctor Manuel Blanco, Lady Ramírez, Daniel Garcerant, Adriana Cruz, Noah Craft, Nancy Gore Saravia

**Affiliations:** 1 Centro Internacional de Entrenamiento e Investigaciones Médicas (CIDEIM), Cali, Colombia; 2 Division of Molecular Medicine, Department of Medicine, Los Angeles Biomedical Research Institute at Harbor-UCLA Medical Center, Torrance, California, United States of America; Institut Pasteur de Tunis, Tunisia

## Abstract

**Background:**

The inflammatory response is prominent in the pathogenesis of dermal leishmaniasis. We hypothesized that regulatory T cells (Tregs) may be diminished in chronic dermal leishmaniasis (CDL) and contribute to healing during treatment.

**Methodology/Principal Findings:**

The frequency and functional capacity of Tregs were evaluated at diagnosis and following treatment of CDL patients having lesions of ≥6 months duration and asymptomatically infected residents of endemic foci. The frequency of CD4^+^CD25^hi^ cells expressing Foxp3 or GITR or lacking expression of CD127 in peripheral blood was determined by flow cytometry. The capacity of CD4^+^CD25^+^ cells to inhibit *Leishmania*-specific responses was determined by co-culture with effector CD4^+^CD25^−^ cells. The expression of *FOXP3*, *IFNG*, *IL10* and *IDO* was determined in lesion and leishmanin skin test site biopsies by qRT-PCR. Although CDL patients presented higher frequency of CD4^+^CD25^hi^Foxp3^+^ cells in peripheral blood and higher expression of *FOXP3* at leishmanin skin test sites, their CD4^+^CD25^+^ cells were significantly less capable of suppressing antigen specific-IFN-γ secretion by effector cells compared with asymptomatically infected individuals. At the end of treatment, both the frequency of CD4^+^CD25^hi^CD127^−^ cells and their capacity to inhibit proliferation and IFN-γ secretion increased and coincided with healing of cutaneous lesions. *IDO* was downregulated during healing of lesions and its expression was positively correlated with *IFNG* but not *FOXP3*.

**Conclusions/Significance:**

The disparity between CD25^hi^Foxp3^+^ CD4 T cell frequency in peripheral blood, Foxp3 expression at the site of cutaneous responses to leishmanin, and suppressive capacity provides evidence of impaired Treg function in the pathogenesis of CDL. Moreover, the concurrence of increased *Leishmania*-specific suppressive capacity with induction of a CD25^hi^CD127^−^ subset of CD4 T cells during healing supports the participation of Tregs in the resolution of chronic dermal lesions. Treg subsets may therefore be relevant in designing immunotherapeutic strategies for recalcitrant dermal leishmaniasis caused by *Leishmania (Viannia)* species.

## Author Summary

The immune inflammatory response is a double edged sword. During infectious diseases, regulatory T cells can prevent eradication of the pathogen but can also limit inflammation and tissue damage. We investigated the role of regulatory T cells in chronic dermal leishmaniasis caused by species of the parasite *Leishmania* that are endemic in South and Central America. We found that although individuals with chronic lesions have increased regulatory T cells in their blood and at skin sites where immune responses to *Leishmania* were taking place compared to infected individuals who do not develop disease, their capacity to control the inflammatory response to *Leishmania* was inferior. However, healing of chronic lesions at the end of treatment was accompanied by an increase in the number and capacity of regulatory T cells to inhibit the function of effector T cells that mediate the inflammatory response. Different subsets of regulatory T cells, defined by the expression of molecular markers, were identified during chronic disease and healing, supporting the participation of distinct regulatory T cells in the development of disease and the control of inflammation during the healing response. Immunotherapeutic strategies may allow these regulatory T cell subsets to be mobilized or mitigated to achieve healing.

## Introduction

Dermal leishmaniasis (DL) caused by species from the *Viannia* subgenus is characterized by a paucity of parasites in lesions associated with a robust inflammatory response and frequently follows a chronic course [1]. Both cutaneous and muco-cutaneous presentations of chronic dermal leishmaniasis (CDL) caused by *Leishmania (Viannia)* are associated with elevated cellular immune responses [2], [3]. Human studies and a recent murine model of chronic dermal disease have shown that a mixed Th1/Th2 cytokine pattern occurs in CDL caused by *L. (Viannia)*, with prominent secretion of IFN-γ, IL-13, IL-10 and TNF-α [4]–[6]. Even though regulatory mechanisms are likely to have a significant role in the development of such an immune response, little is known about their impact in determining susceptibility and shaping disease outcome.

Regulatory T cells (Tregs) maintain tolerance to self tissues by inhibiting the action of auto-antigen reactive lymphocytes in an antigen-specific manner. In infectious diseases, Tregs also regulate the intensity and duration of immune responses, limiting damage to self tissues [7]–[8]. In the murine model of DL caused by *L. major*, Tregs have been shown to promote parasite persistence and reactivation in the resistant strain C57Bl/6 while inhibiting pathology in the susceptible Balb/c strain [9]–[12]. Tregs also suppressed pathology in mouse strains susceptible to disease caused by *L. amazonensis* [13]. These observations indicate that regulation of *Leishmania*-specific responses by Tregs may differentially alter the outcome of infection according to the susceptibility phenotype dictated by the host response. In resistant individuals, excessive Treg action may interfere with elimination of infection, whereas in susceptible individuals deficient Treg function may lead to excessive inflammation and dermal pathology.

Studies in humans infected with species from the *Viannia* subgenus have demonstrated that T cells with regulatory phenotype and function are present in cutaneous lesions [14]–[17]. An association between increased Foxp3 expression and unresponsiveness to treatment and chronic disease has been reported in human DL caused by *L. guyanensis* infection [16]–[17]. In contrast, no differences were found in the frequency of Tregs in peripheral blood between asymptomatically infected individuals (AI) and patients with CDL in *L. braziliensis* infection [14]. Hence, the role of Tregs in the pathogenesis of DL and their participation in the therapeutic response remain unclear.

The purpose of this study was to evaluate the role of Tregs in CDL caused by species of the *Viannia* subgenus and in the resolution of chronic lesions following treatment with pentavalent antimony. We found that lack of regulation of IFN-γ secretion by Tregs was associated with development of chronic disease, while an increase of Treg function after treatment was associated with lesion healing.

## Methods

### Experimental Strategy and Rationale

In this study, asymptomatic infection was considered to approximate clinical resistance to natural infection, and chronic disease to define a clinically susceptible phenotype, analogous to the healing and non-healing phenotypes in murine models of cutaneous leishmaniasis. Because AI and DL patients evidently remain infected indefinitely [18]–[23], exposure to *Leishmania* antigens would persist in both clinical outcomes. Since DL is generally a self-resolving disease, discrimination of spontaneous healing and chronic disease is not reliably determined during early or intermediate stages of evolution. However, longer times of evolution (chronic disease) have been shown to be associated with increased immune reactivity to *Leishmania* antigens including significantly higher antibody titers and DTH responses [2]. Furthermore, Th1/Th2 transcription factor expression and inflammatory cytokine responses distinguished asymptomatic clinical outcome and chronic cutaneous disease [5]. The rationale for analyzing the Treg response in asymptomatic infection and chronic disease was, therefore, that these phenotypically distinguishable outcomes are natural expressions of clinical resistance and susceptibility to human dermal leishmaniasis.

### Ethics Statement

All participants provided written informed consent. The study protocol, consent forms and all procedures were approved by the CIDEIM Institutional Review Board for the ethical conduct of research involving human subjects.

### Human Subjects

Participants were residents of endemic areas for *L. panamensis* and *L. braziliensis* located within the southwestern Pacific coast region of Colombia (Departments of Valle del Cauca and Nariño) [24]. We included cases caused by both of these species of the *Viannia* subgenus because there is significant overlap of clinical presentations and broad measures of immune responses (DTH, lymphocyte proliferation and antibody titer) in patients with dermal disease caused by these species [2], [25]. AI had a positive LST and no evidence or history of dermal lesions. CDL patients had dermal lesions of ≥6 months duration, parasitological diagnosis by microscopic examination of tissue samples from lesions, culture or biopsy, and had not received anti-leishmanial treatment before enrollment. All subjects had negative serology for HIV and HTLV-1. A LST was performed in all participants and evaluated at 48 hours as previously described [26]. Leishmanin (Instituto Nacional de Salud, Colombia) was composed of equal amounts of *L. panamensis* and *L. amazonensis* promastigote proteins at a concentration of 5 µg/mL. Peripheral blood samples (100 mL), and skin biopsies from a leishmaniasis lesion and LST reaction site were obtained upon entry into the study. The species of parasite strains isolated from patients with a positive culture were determined as previously described [27]. CDL patients were treated with meglumine antimoniate at a dose of 20 mg Sb/kg/day. After treatment, a second 100 mL blood sample and a biopsy from the same lesion site were obtained and clinical responses were evaluated. Complete healing was defined as total re-epithelialization and absence of any evidence of inflammation for all lesions. Partial healing was estimated as the percentage of reduction of ulcer/plaque area or nodule volume. Skin biopsies from four healthy volunteers were obtained for normalization of gene expression data. Biopsies were obtained using a 4 mm disposable punch, embedded in OCT, frozen and stored in liquid nitrogen until processed.

### Cell Isolation

PBMCs were isolated by centrifugation over Histopaque-1077 (Sigma-Aldrich, St. Louis, MO). CD4^+^CD25^−^ and CD4^+^CD25^+^ cells were isolated by MACS using the CD4^+^CD25^+^ Regulatory T cell isolation kit (Miltenyi Biotec, Bergisch-Gladbach, Germany) following the manufacturer's instructions. Purity assessed by staining with anti-CD4 and anti-CD25 was ≥90% for both populations. Monocytes were isolated by allowing PBMCs to adhere to plastic for 2 hours followed by three washes with PBS. Purity assessed by staining with anti-CD14 was ≥65% and contaminating CD4^hi^ lymphocytes were <5%.

### Evaluation of Cell Marker Expression

PBMCs from 12 AI, 14 CDL patients before treatment, and 11 CDL patients after treatment were suspended in FACS buffer (1× PBS, 1% BSA, 0.1% NaN_3_) and incubated for 30 minutes with anti-CD4-APC (BD Biosciences, San Jose, CA), anti-CD25-PE (Miltenyi Biotec) and anti-CD127-FITC (eBioscience, San Diego, CA) or anti-CD4-FITC (BD Biosciences), anti-glucocorticoid-induced TNF receptor family-related protein (GITR)-PE and anti-CD25-APC (eBioscience). For evaluation of forkhead box p3 (Foxp3), cells were stained with anti-CD4-FITC and anti-CD25-APC, fixed and permeabilized using the Fixation and Permeabilization kit (eBiosciences), and incubated with anti-Foxp3-PE or anti-Rat IgG2a-PE (eBioscience). After washing, cells were analyzed in a Navios flow cytometer (Beckman Coulter, Brea, CA) and data was analyzed using FlowJo 7.6 software (Tree Star, Inc., Ashland, OR). Gates used for analysis were set using isotype controls.

### Co-Culture of CD4^+^CD25^−^ and CD4^+^CD25^+^ Cells

Killed promastigotes of the *L. panamensis* strain MHOM/COL/81/L13 were prepared by the freeze/thaw method, as previously described [28]. CD4^+^CD25^−^ cells from 12 AI, 14 CDL patients before treatment, and 11 CDL patients after treatment were incubated with 5 µM carboxyfluorescein diacetate succinimidyl diester (CFSE, Invitrogen, Carlsbad, CA) for 10 minutes at 4°C, washed three times with PBS and resuspended in RMPI 1640 (Sigma-Aldrich) with 10% FBS (Gibco, Carlsbad, CA), 2 mM L-glutamine, penicillin (100 U/mL) and streptomycin (100 mg/mL). 1×10^6^ CD4^+^CD25^−^ cells per well were distributed in 24 well plates with 2×10^5^ *L. panamensis* promastigotes, 5×10^5^ monocytes from the same subject (used as antigen presenting cells, APCs), or phytohaemagglutinin (PHA, Sigma-Aldrich) 10 µg/mL, and CD4^+^CD25^+^ cells at 1∶0, 4∶1 or 1∶1 CD4^+^CD25^−^: CD4^+^CD25^+^ cell ratios, in a final volume of 1 mL. After 5 days of incubation at 37°C with 5% CO_2_, cells and supernatants were harvested for evaluation of proliferation and cytokine secretion, respectively. Cells were stained with anti-CD4-APC and analyzed by flow cytometry. CFSE fluorescence in CD4^+^CD25^−^ cells was evaluated in the CD4^+^ gate. Unlabeled CD4^+^CD25^+^ cells and CD4^+^ lymphocytes contaminating the APC preparation were gated out of the analysis (Figure S1A). Regions were drawn for each proliferation peak induced by PHA (Figure S1B) and the proliferation index (PI) was calculated with the formula PI = Σ(% in region×2^n−1^), where n is the region number [29]. Interferon-γ (IFN-γ) and interleukin-10 (IL-10) were measured in supernatants by ELISA as previously described [28]. Co-cultures were considered to have positive proliferation or IFN-γ secretion when the value in wells with CD4^+^CD25^−^ cells, APCs and *L. panamensis* or PHA was higher than the mean+2SD of the control wells without APCs, *L. panamensis* or neither. The percent inhibition of proliferation or IFN-γ secretion by CD4^+^CD25^+^ cells was calculated in positive co-cultures using the formula: (value without CD4^+^CD25^+^ cells - value with CD4^+^CD25^+^ cells)/(value without CD4^+^CD25^+^ cells) ×100. Negative results were considered 0% inhibition.

### Real-Time PCR Analysis

RNA was isolated from LST biopsies from 5 AI and 7 CDL patients and lesion biopsies from 11 CDL patients both before and after treatment using the RNeasy mini kit (QIAGEN, Valencia, CA) according to the manufacturer's instructions. For cDNA synthesis, 100 ng total RNA was transcribed with the High-capacity cDNA reverse transcription kit (Applied Biosystems, Foster City, CA), following the manufacturer's instructions. Gene expression was measured in real-time with the CFX966 Real Time System (Bio-Rad, Hercules, CA) using Taqman Universal PCR master mix and Taqman gene expression assays for *GAPDH* (Hs99999905m-1), *FOXP3* (Hs01085835-m1), *IFNG* (Hs00174143-m1), *IL10* (Hs00174086-m1) and *IDO* (Hs00158027-m1) genes (Applied Biosystems), following the manufacturer's instructions. The expression levels of *FOXP3*, *IFNG*, *IL10* and *IDO* relative to healthy skin were calculated using the ΔΔCT method, as previously described [30], using *GAPDH* as endogenous reference gene and four healthy skin biopsies as calibrators.

### Statistical Analysis

The Kolmogorov-Smirnov test was used to determine parametric or non-parametric distribution of the data. Thereafter, for comparisons between AI and CDL patients, parametric data were analyzed using student t-test, and the Mann-Whitney test was applied for non-parametric data. For comparisons between CDL patients before and after treatment, parametric and non-parametric data were analyzed using the paired t-test and the Wilcoxon signed-rank test, respectively. Correlation analyses were conducted using the Spearman coefficient. Statistical significance was defined as p<0.05. All data were analyzed using Prism 5 software (GraphPad Software, Inc., La Jolla, CA).

## Results

### Clinical Characteristics of the Study Subjects

Twelve AI and 14 CDL patients participated in this study. There were no significant differences in age, gender or size of LST reaction between AI and CDL patients (data not shown). The clinical characteristics of CDL patients are summarized in Table 1. Three patients were lost to follow-up. The other eleven patients were evaluated at a second visit within 24 days of completing treatment. Five patients showed complete healing, and lesions in six patients had substantially improved though not completely healed (Table 1).

**Table 1:** Clinical characteristics and evolution of chronic dermal leishmaniasis patients.

Subject code	Isolated parasite	Lesion duration (months)	Lesion number	Lesion Type	Time of second sample (Days after treatment)	% Cured^a^
111	ND	8	1	Nodule	2	50
112	*L. braziliensis*	6	2	Ulcers	1	100
113	*L. panamensis*	7	1	Ulcer	Lost to followup	NA
114	*L. panamensis*	6	3	Ulcers	Lost to followup	NA
116	*L. panamensis*	6	5	Ulcers/plaque/mucosal	1	80
117	*L. panamensis*	18	8	Plaques/papules/mucosal	24	100
118	*L. panamensis*	6	1	Ulcer	Lost to followup	NA
121	*L. panamensis*	20	3	Plaques/Nodule	1	90
209	ND	6	1	Ulcer	5	100
214	ND	24	1	Ulcer	2	60
216	*L. braziliensis*	240	1	Ulcer	2	80
217	*L. panamensis*	36	1	Plaque	2	100
218	ND	48	1	Ulcer	2	80
223	ND	6	3	Plaques	23	100

a % of surface area of ulcer(s) or plaque(s) healed or volume of nodule(s) reduced at the end of treatment; ND: not determined because culture was not necessary or no strain was obtained; NA: not applicable.

### CDL Patients Presented a Higher Frequency of CD4^+^CD25^hi^Foxp3^+^ Cells in Peripheral Blood than AI

**Figure 1:**
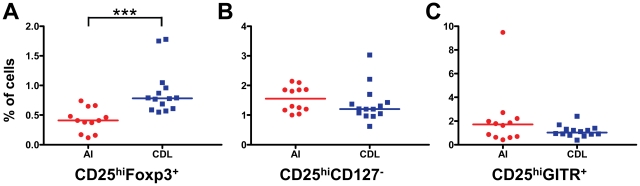
CDL patients have a higher frequency of CD4^+^CD25^hi^FoxP3^**+**^ cells in peripheral blood than AI. PBMCs from AI (n = 12) and CDL patients (n = 14) were stained for CD4, CD25 and either Foxp3, CD127, or GITR and analyzed by flow cytometry. **A**. Frequency of CD4^+^CD25^hi^Foxp3^+^ cells. **B**. Frequency of CD4^+^CD25^hi^CD127^−^ cells. **C**. Frequency of CD4^+^CD25^hi^GITR^+^ cells. ***p<0.001, Mann-Whitney test. The median is represented by a horizontal line.

High expression of CD25 has been described as a reliable marker for Tregs [31]–[34]. Lack of expression of CD127, expression of GITR, or expression of Foxp3 have also been described as Treg markers [34]–. Therefore, to evaluate the frequency of Tregs in the peripheral blood of AI and CDL patients, we measured the frequency of CD25^hi^ CD4 T cells that expressed each of these markers. We found that CD4^+^CD25^hi^Foxp3^+^ cells were more abundant in the peripheral blood of CDL patients (0.9±0.4% in CDL patients vs. 0.4±0.2 in AI, p = 0.0003; Figure 1A), while significant differences were not observed in the frequency of CD4^+^CD25^hi^CD127^−^ and CD4^+^CD25^hi^GITR^+^ cells between the two groups (Figures 1B and C).

### CD4^+^CD25^+^ Cells from AI Demonstrated a Higher Capacity to Suppress CD4 Effector T Cell IFN-γ Secretion than Those from CDL Patients

**Figure 2:**
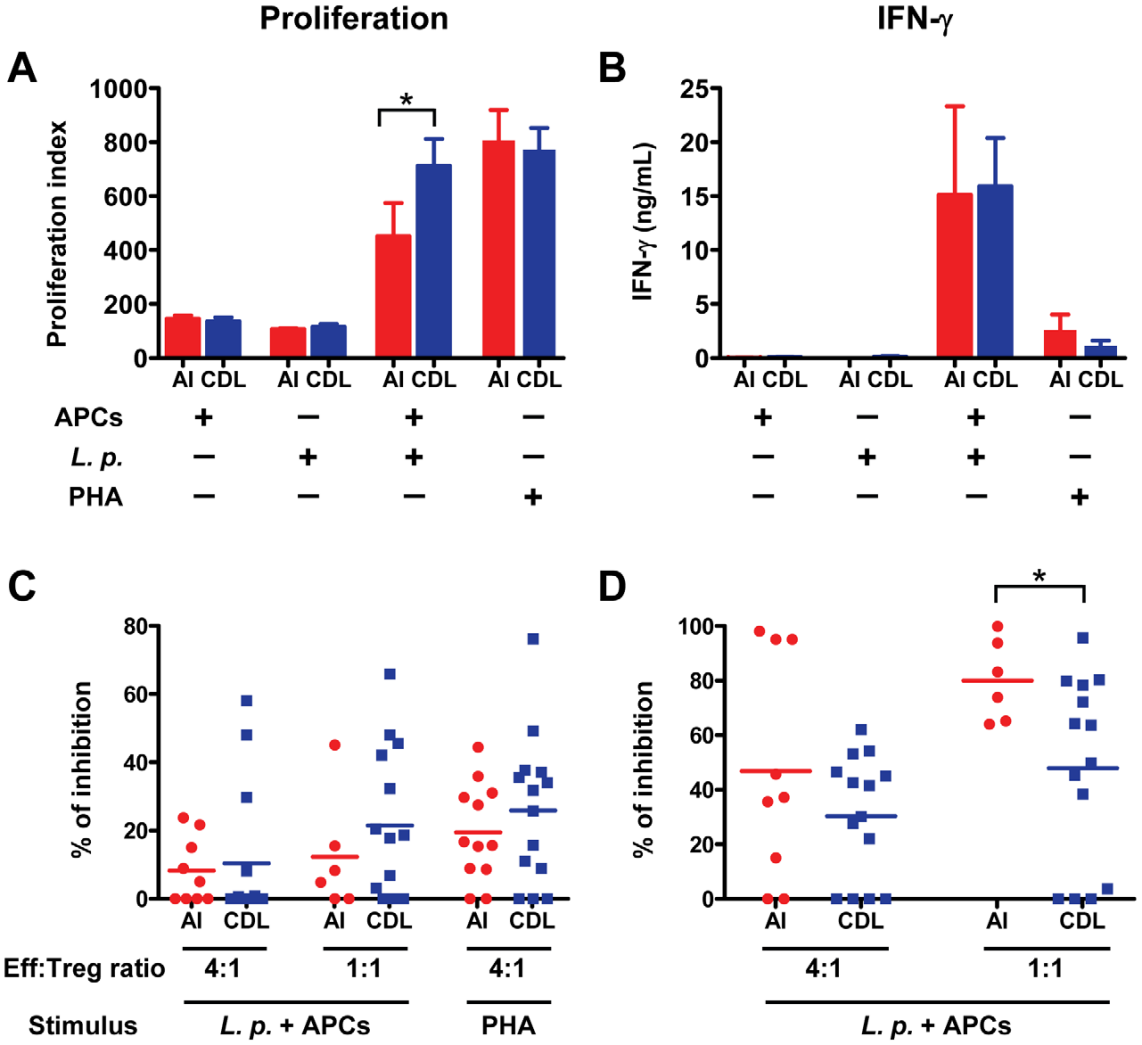
CD4^**+**^CD25^**+**^ cells from AI have a higher capacity to suppress IFN-γ secretion by effector cells. CD4^+^CD25^−^ (effector) cells from AI (n = 12) and CDL patients (n = 14) were cultured for 5 days with antigen presenting cells (APCs), *L. panamensis* (*L.p.*), or both, or with PHA, as indicated. **A**. Proliferation of CD4^+^CD25^−^ cells. **B**. IFN-γ levels secreted by CD4^+^CD25^−^ cells. Positive proliferation and IFN-γ secretion were defined as levels above the mean+2SD of control wells from the same donor. For subjects whose effector cells showed positive proliferation and IFN-γ secretion (n = 9 for AI and n = 14 for CDL patients, *L.p.*+APCs stimulation; n = 12 for AI and n = 14 for CDL patients, PHA stimulation), cultures were conducted in the presence or absence of CD4^+^CD25^+^ (regulatory) cells. **C**. Inhibition of CD4^+^CD25^−^ cell proliferation induced by *L.p.*+APCs or PHA by CD4^+^CD25^+^ cells. **D**. Inhibition of CD4^+^CD25^−^ cell IFN-γ secretion induced by *L.p.*+APCs by CD4^+^CD25^+^ cells. 1∶1 effector∶Treg ratio could not be tested in 3 AI because insufficient numbers of CD4^+^CD25^+^ cells were obtained (n = 6 for AI and n = 14 for CDL patients); *p<0.05, Mann-Whitney test. A and B show means with SEM. C and D show the mean as a horizontal line.

We evaluated the functional capacity of Tregs to suppress *Leishmania*-specific proliferation and IFN-γ secretion using co-cultures of CD4^+^CD25^−^ cells (effectors) and CD4^+^CD25^+^ cells (Tregs) from peripheral blood. In the absence of Tregs, proliferation and IFN-γ secretion were induced by *L. panamensis* antigens in effector T cells from all CDL patients and 9 of 12 AI. Proliferation was significantly higher for CDL patients in relation to AI (p = 0.037, Figure 2A), while no statistically significant differences were detected for IFN-γ secretion (Figure 2B). No significant effector functions were observed in the absence of either APCs or *L. panamensis* antigens (Figures 2A and B), indicating that they were the result of antigen-specific APC-T cell interactions. *L. panamensis* induced similar levels of proliferation and IFN-γ secretion in patients infected with *L. panamensis* or *L. braziliensis* and in patients in which infecting species was undetermined (data not shown), confirming recall of T cell responses across species. High levels of proliferation were induced by PHA in all subjects (Figure 2A), while IFN-γ secretion was low with this stimulus under the conditions of assay (Figure 2B), being positive only for 6 AI and 3 CDL patients.

Suppression assays with *L. panamensis* stimulation were performed at effector∶Treg ratios of 4∶1 and 1∶1 in subjects that had positive effector functions, except for three AI from whom the number of CD4^+^CD25^+^ cells isolated was insufficient to evaluate the 1∶1 ratio. At a 4∶1 effector∶Treg ratio, no statistically significant differences in antigen-specific suppression were detected between AI and CDL patients (Figures 2C and D). At a 1∶1 effector∶Treg ratio, suppression of IFN-γ secretion was significantly higher in AI compared to CDL patients (80±6.1% vs. 48±9.2%, p = 0.044; Figure 2D) whereas suppression of proliferation was not significantly different between the two groups (Figure 2C). To evaluate suppression after a polyclonal stimulus, inhibition of proliferation by Tregs at a 4∶1 ratio was evaluated after PHA stimulation. No significant differences in suppression of proliferation induced by PHA were detected between AI and CDL patients (mean inhibition 19.5±4.1% and 25.9±5.8%, respectively; p = 0.313; Figure 2C).

### *FOXP3* Expression Is Higher in LST Sites of CDL Patients than AI

**Figure 3:**
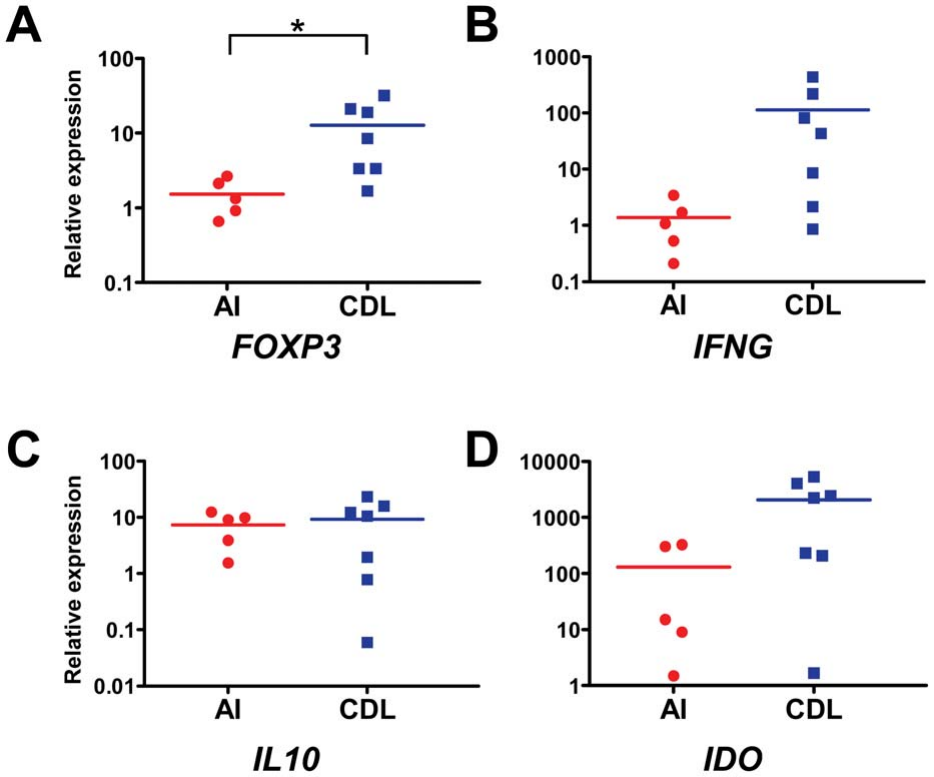
Relative expression of *FOXP3* is higher in leishmanin skin test (LST) sites from CDL patients. RNA was isolated from biopsies of LST sites from AI (n = 5) and CDL patients (n = 7) and the expression of *FOXP3* (**A**), *IFNG* (**B**), *IL10* (**C**) and *IDO* (**D**) was measured by qRT-PCR. The expression of each gene relative to healthy skin of four normal controls was calculated using the ΔΔCT method. *p<0.05, unpaired t-test. The median is represented by a horizontal line.

We next examined the influence of Tregs in situ by measuring the transcription of genes related to immune regulation and inflammation at the site of injection of leishmanin antigen from 5 AI and 7 CDL patients. We evaluated the relative expression of four genes: *FOXP3*, *IFNG*, *IL10*, and 2,3 indoleamine deoxygenase (*IDO*), an enzyme expressed by APCs that is involved in immune regulation through the catabolism of the essential aminoacid tryptophan [38], [39]. We found that the relative expression of *FOXP3* was significantly higher in CDL patients in relation to AI (relative expression 12.7±4.3 vs. 0.7±0.8, p = 0.046, Figure 3A). No statistically significant differences were found for the other genes, although several individuals with chronic disease showed upregulation of *IFNG* and *IDO* (Figures 3B–D).

### CD4^+^CD25^hi^CD127^−^ Cells and Suppression by CD4^+^CD25^+^ Cells Increased after Treatment of CDL

**Figure 4:**
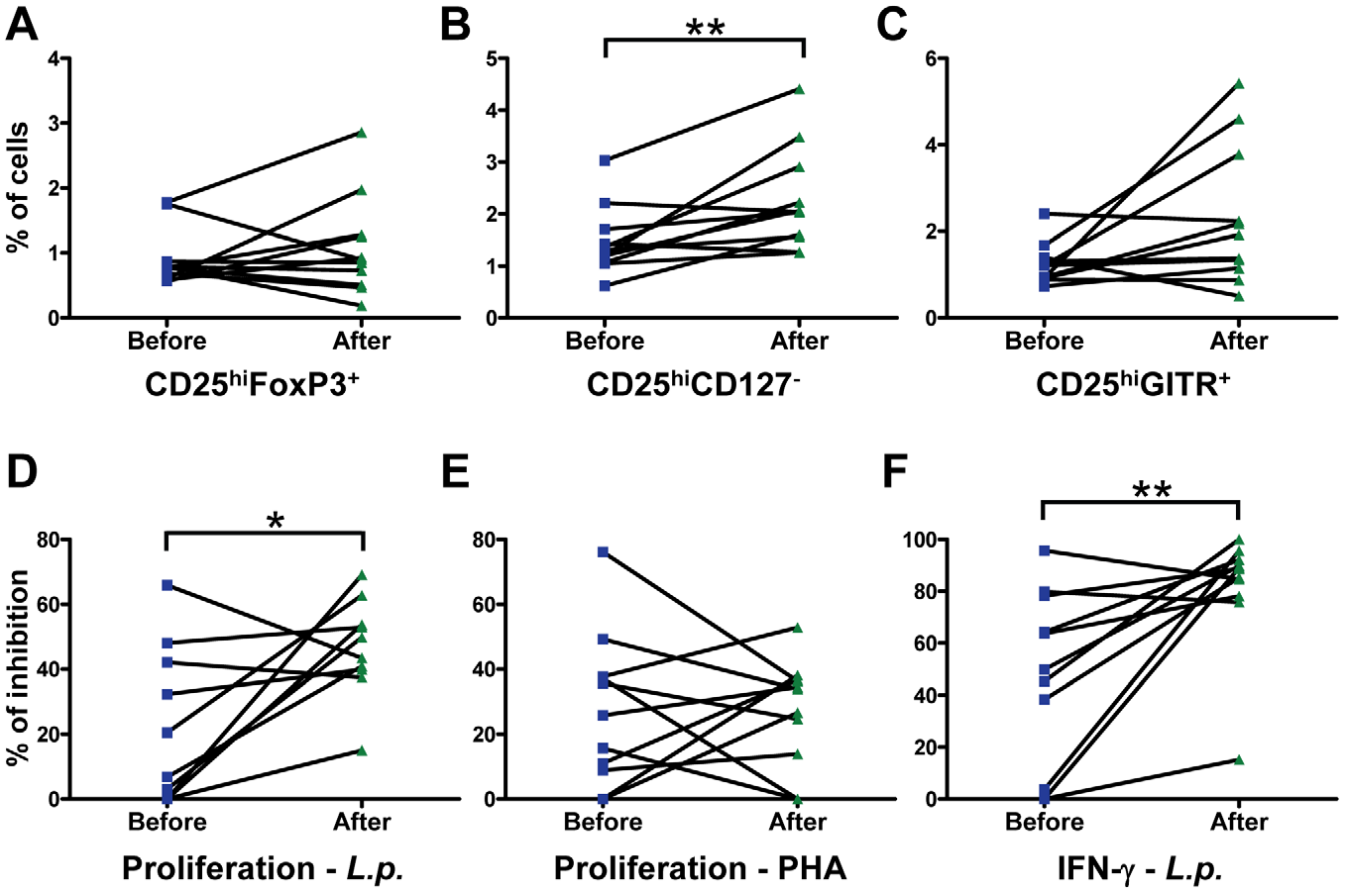
Treg frequency and function increase after treatment of CDL. Frequency of cells with a Treg phenotype in PBMCs and suppressive capacity of CD4^+^CD25^+^ cells were determined for CDL patients (n = 11) before and after treatment. **A**. Frequency of CD4^+^CD25^hi^Foxp3^+^ cells. **B**. Frequency of CD4^+^CD25^hi^CD127^−^ cells. **C**. Frequency of CD4^+^CD25^hi^GITR^+^ cells. **D**. Inhibition of CD4^+^CD25^−^ cell proliferation by CD4^+^CD25^+^ cells at a 1∶1 ratio after stimulation with *L. panamensis (L.p.)* and APCs. **E**. Inhibition of CD4^+^CD25^−^ cell proliferation by CD4^+^CD25^+^ cells at a 4∶1 ratio after stimulation with PHA. **F**. Inhibition of CD4^+^CD25^−^ cell IFN-γ secretion by CD4^+^CD25^+^ cells at a 1∶1 ratio after stimulation with *L.p.* and APCs. Inhibition was calculated only for subjects that had proliferation and IFN-γ levels above the average+2SD of control wells from the same co-culture (n = 10 for proliferation and 11 for IFN-γ secretion with *L.p.* stimulation and n = 11 for PHA stimulation). *p<0.05, paired t-test, **p<0.01, Wilcoxon signed-rank test.

At the end of treatment of CDL, the proportion of CD4^+^CD25^hi^CD127^−^ cells increased significantly, from 1.5±0.2% to 2.3±0.3% (p = 0.0009, Figure 4B), whereas the frequency of CD4^+^CD25^hi^Foxp3^+^ and CD4^+^CD25^hi^GITR^+^ cells did not change significantly (Figure 4A and C). Proliferation and IFN-γ secretion in the absence of Tregs did not change significantly at the end of treatment, although significant proliferation was no longer observed in one patient (data not shown). In the presence of Tregs at a 1∶1 effector∶Treg ratio, 8 of 10 CDL patients demonstrated increased inhibition of proliferation by CD4^+^CD25^+^ cells at the end of treatment and 9 of 11 showed an increase in inhibition of IFN-γ secretion (Figures 4D and F). Inhibition of both parameters increased significantly for the group as a whole, with the mean percent inhibition of proliferation increasing from 21.9% to 46.5% (p = 0.025, Figure 4D) and the mean inhibition of IFN-γ secretion rising from 47.2% to 81.3% (p = 0.007, Figure 4F). Suppression of proliferation induced by PHA did not change significantly after treatment (p = 0.99; Figure 4E).

### Expression of *IDO* in CDL Lesions Decreased after Treatment and Was Correlated with *IFNG* Expression

**Figure 5:**
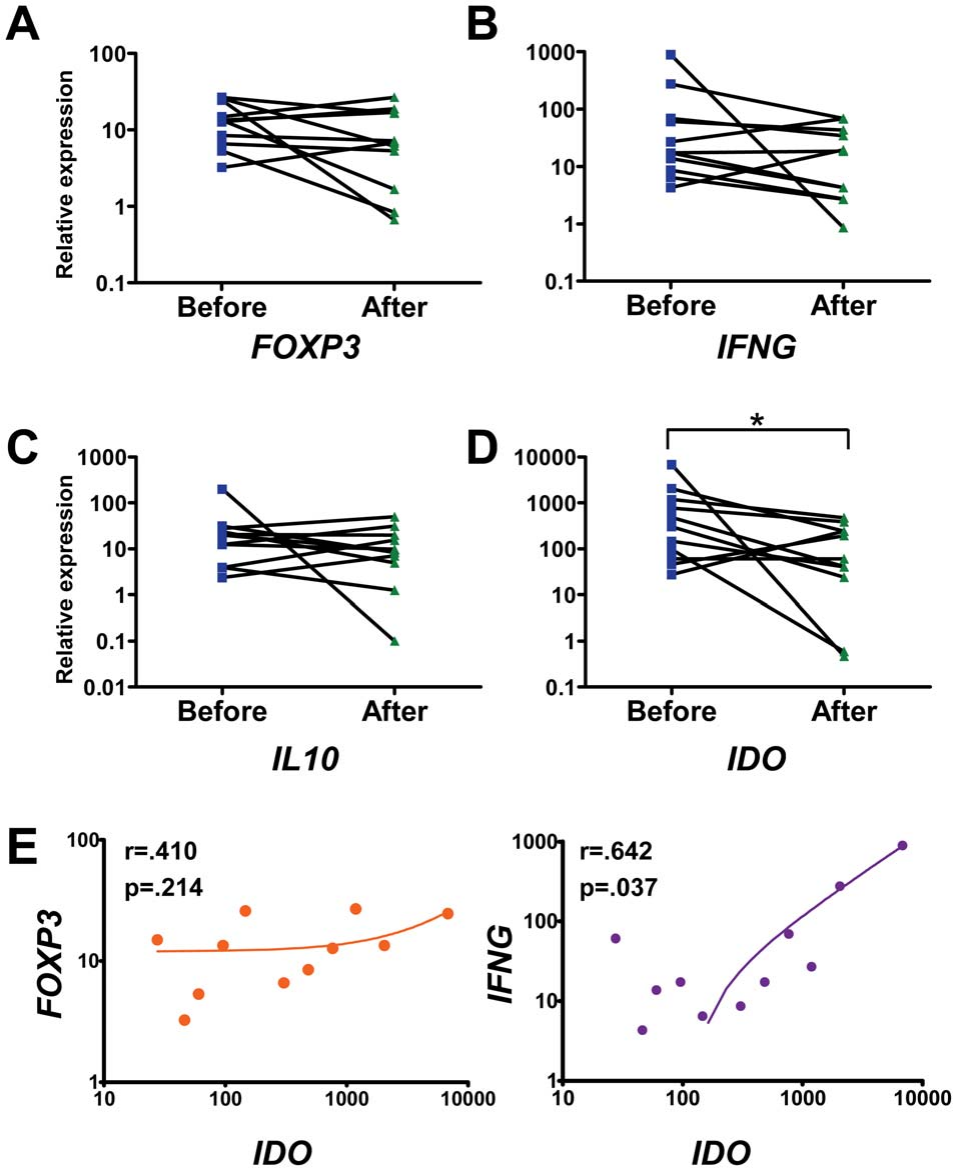
Relative expression of *IDO* decreases in CDL patients after treatment and is correlated with *IFNG*. RNA was isolated from biopsies of lesions from CDL patients (n = 11) before and after treatment and the expression of *FOXP3* (**A**), *IFNG* (**B**), *IL10* (**C**) and *IDO* (**D**) was measured by qRT-PCR. The expression of each gene relative to healthy skin of four normal controls was calculated using the ΔΔCT method. **E**. Correlation between the relative expression of *IDO* and *FOXP3* (left panel) or *IFNG* (right panel). *p<0.05, Wilcoxon signed-rank test.

The relative expression of *FOXP3* and *IL10* did not change significantly after treatment of CDL (Figures 5A and C). The relative expression of *IFNG* decreased in 8 out of 11 patients at the end of treatment, with the mean value declining from 127.0±80.3 to 24.3±7.9. However, this difference was not statistically significant (p = 0.148, Figure 5B). Finally, although the expression of *IDO* in chronic lesions at the start of the study varied widely between individuals, at the end of treatment the expression of this gene was significantly downregulated from 1087±601 to 154±50 (p = 0.037, Figure 5D). Because IDO expression in APCs is upregulated by both inflammatory signals (particularly IFN-γ) [40], [41] and Tregs (through CTLA4-B7 interactions) [42], we examined whether *IDO* expression was correlated with that of *IFNG* or *FOXP3* in CDL lesions. Our analysis revealed a significant positive correlation between *IFNG* and *IDO* expression. In contrast, *FOXP3* expression was not significantly correlated with *IDO* expression (Figure 5E). Thus, IDO expression in the skin of CDL patients was most likely upregulated by IFN-γ.

### Suppression in Co-Cultures of Effector CD4 T Cells and Tregs Was Not Mediated by IL-10

**Figure 6:**
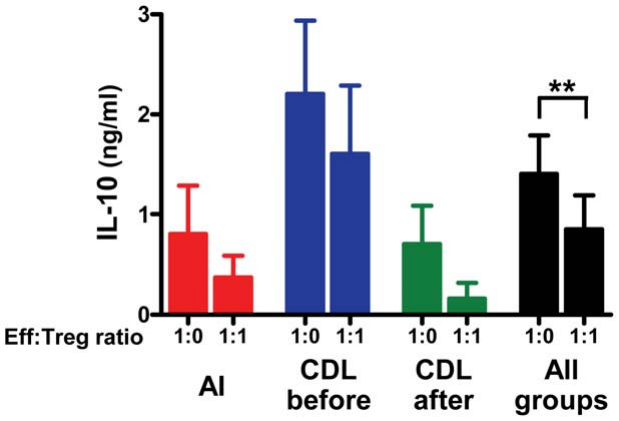
IL-10 does not mediate suppression of effector functions by CD4^+^CD25^+^ cells. IL-10 was measured in supernatants from the CD4^+^CD25^−^-CD4^+^CD25^+^ co-cultures by ELISA. The level of IL-10 in the absence (1∶0 ratio) or presence (1∶1 ratio) of CD4^+^CD25^+^ cells is shown for AI (n = 6), CDL patients before treatment (n = 14), CDL patients after treatment (n = 11) and all co-cultures combined (n = 31). **p<0.01, Wilcoxon signed-rank test. Means with SEM are shown.

To determine whether suppression of effector functions was attributable to IL-10 secretion by CD4^+^CD25^+^ cells, we evaluated IL-10 in the supernatants of the effector-Treg co-cultures. The presence of CD4^+^CD25^+^ cells in the co-cultures did not result in higher IL-10 concentrations in any of the study groups (Figure 6). To the contrary, analysis of co-cultures at the 1∶1 ratio (n = 31) for all groups revealed significantly lower IL-10 secretion in the presence of CD4^+^CD25^+^ cells (Figure 6). Therefore, inhibition of effector CD4 T cell functions in this in vitro system was not attributable to IL-10 secretion by Tregs. Rather, these results indicate that IL-10 secretion by CD4^+^CD25^−^ effector T cells and/or APCs was also inhibited by Tregs.

## Discussion

The regulation of adaptive immune responses is indispensable for the effective clearance of antigen without harm to self tissues. Although the importance of Tregs in this process is well established, evaluation in human disease is challenging because of the increasing recognition of the complexity of their phenotype and function. To clarify the role of Tregs in the pathogenesis and healing of DL caused by species of the *Viannia* subgenus, we studied a cohort of subjects from an area in Colombia endemic for *L. panamensis* and *L. braziliensis*. Comparisons of the frequency and suppressive function of cells expressing Treg markers in peripheral blood and the expression of genes related to inflammation and regulation in the skin of AI and CDL patients, and CDL patients before and after treatment, yielded evidence that Tregs participate in clinical resistance/susceptibility and lesion healing.

Patients with chronic dermal disease had a significantly higher frequency of CD25^hi^FoxP3^+^ CD4 T cells in their peripheral blood than infected individuals who did not develop disease. Additionally, *FOXP3* expression was upregulated at skin sites of challenge with leishmanin antigen only in CDL patients. These results are consistent with several examples of chronic disease in which Foxp3 is upregulated in T cells [43]–[46] and previous reports of infiltration of dermal lesions caused by *L. guyanensis* and *L. brazilienisis* by Foxp3^+^ cells [15], [16], [47] and upregulation of *FOXP3* in lesions from post Kala Azar DL [48]. Foxp3 may therefore constitute a marker for chronic DL caused by *L. (Viannia)* species.

Although CDL patients had higher numbers of CD25^hi^Foxp3^+^ cells in peripheral blood than AI, the capacity of their CD4^+^CD25^+^ cells to inhibit pro-inflammatory IFN-γ secretion by effector T cells elicited by *Leishmania* antigens was lower. This suggests that the CD25^hi^Foxp3^+^ cells that accounted for the higher frequency in CDL patients are not specific for *Leishmania* antigens. Since Foxp3 is a marker of natural Tregs generated in the thymus after interaction with self antigens [37], [51], these cells may be a subset of Tregs specific for self antigens that are released during the inflammatory response [7], [52], [53]. Alternatively, the higher proportion of CD25^hi^Foxp3^+^ cells may reflect chronic activation in CDL patients, since FoxP3 can be transiently expressed by activated T cells [49], [50]. However, we do not favor this explanation because high expression of CD25 has been consistently proven to be a marker of Tregs [31]–[34] and effector cells expressing FoxP3 are mostly not CD25^hi^ [50]. Thus, we postulate that CD25^hi^Foxp3^+^ Tregs, although more frequent in CDL, are not responsible for the antigen specific suppression demonstrated in the co-culture assays. Rather, this suppression is attributable to a *Leishmania*-specific, Foxp3^−^ subset. However, we cannot rule out the possibility that *Leishmania*-specific effector cells acquired resistance to regulation by Tregs after chronic antigenic stimulation in CDL.

Clinical evaluation at the end of treatment with pentavalent antimony showed that all patients had initiated a healing response. Half had already healed completely while the other half showed significant improvement (≥50%, Table 1). Clinical follow-up was possible for 7/11 patients at ≥13 weeks after starting treatment and confirmed that all seven had completely healed (data not shown). Thus, no evidence of treatment failure was seen in this study cohort. The healing responses in this CDL cohort were associated with significant increases in the frequency of CD25^hi^CD127^−^ CD4 T cells in peripheral blood and in *Leishmania*-specific inhibition of CD4 T cell effector functions by CD4^+^CD25^+^ cells. In fact, of the 11 patients, 10 were able to inhibit IFN-γ secretion by ≥75% at the end of treatment and 9 showed an increase in CD4^+^CD25^hi^CD127^−^ cells. Furthermore, transcription of *IFNG* at the lesion site was not significantly upregulated at the end of treatment, but rather decreased in 8 of the 11 patients. Interestingly, we found that Foxp3 expression did not change after treatment in CD4^+^CD25^hi^ blood cells or at the lesion site, suggesting that a Foxp3^+^ subset of Tregs was not responsible for the heightened suppression that was associated with healing. Rather, these findings support the participation of a CD127^−^, *Leishmania*-specific subset of Tregs in the healing of chronic dermal lesions caused by *L. (Viannia)*.

Induction of Tregs during chronic infections is the result of antigen presentation in a particular cytokine environment [7], [53]. In the current study, parasite killing mediated by the anti-leishmanial drug would presumably lead to release of antigens, new antigen presentation events and Treg induction and/or activation in the peripheral lymphoid organs [54]. Newly induced Tregs would home to the lesion site to mitigate the inflammatory response and aid in tissue repair. Homing of Tregs to the skin is essential for the maintenance of skin homeostasis and for suppression of Th1 cell-mediated responses [55], [56]. Furthermore, Tregs have been shown to contribute substantially to tissue repair by providing regulation at sites of healing in many experimental models. In an in vitro wound healing model, Tregs were shown to counteract Th17 cell-mediated inhibition of fibroblast migration into the wound [57], and tissue regeneration after kidney injury was shown to depend on inhibition of pro-inflammatory cytokine secretion by Tregs [58]. In another in vitro model, intact extracellular matrix components that gradually replace inflammation-promoting fragmented components during tissue repair induce Tregs and activate their function [59]. Therefore, healing of dermal lesions such as those present in CDL would likely benefit from immune regulation by Tregs. Since all patients had initiated a healing response at the end of treatment, our results are consistent with the participation of a subset of Foxp3^−^ CD127^−^ *Leishmania*-specific Tregs in lesion resolution.

The limited absolute numbers of CD4^+^CD25^+^ cells allowed us to test only two effector∶Treg ratios. Significant differences in antigen-specific suppression were detected at the 1∶1 ratio, but not at the 4∶1 ratio. Since suppression by Tregs is activated through cognate peptide/MHC-TCR interactions [60], the inability to detect suppression at a higher ratio is probably due to the expected low frequency of *Leishmania*-specific Tregs at the outset of co-culture and the absence of proliferation by these cells [61] (as evidenced by lack of variation in forward scatter, Figure S1A). Therefore, even though the number of *Leishmania*-specific CD25^hi^ T cells in the co-culture is unknown, the observation of significant suppression indicates that the number reached an effective threshold under the assay conditions at a 1∶1 ratio. In addition, the CD4^+^CD25^+^ preparation includes activated effector T cells that upregulate CD25 and, therefore, the number of Tregs in the culture would have been diluted by these cells. For these reasons, we believe that the suppression observed in our system at a 1∶1 ratio reflects the presence of circulating antigen-specific Tregs that can contribute to the mitigation of the inflammatory response upon homing to the lesion site.

We examined the participation of IL-10 in the inhibition of CD4 T cell effector functions in our co-cultures because this cytokine is secreted by T cells with an effector phenotype in intracellular parasitic infections [62]–[64] as well as by Tregs. Furthermore, we have previously determined in a population from the same region that IL-10 is expressed in susceptible individuals and related to both development of CDL and IFN-γ secretion [4]. Hence, it was conceivable that inhibition was due to IL-10 secretion by a CD25^+^ activated effector population present in the CD4^+^CD25^+^ preparation and not by Tregs. The diminished production of IL-10 in co-cultures including CD4^+^CD25^+^ cells demonstrated that IL-10 was not responsible for suppression by these cells, supporting the interpretation that the observed inhibition was indeed mediated by bona fide Tregs. We also found that *IL10* transcription was not significantly changed at the lesion site immediately following treatment, suggesting that this cytokine is not prominent in the healing process.

IDO is an enzyme expressed by APCs that metabolizes tryptophan, an essential aminoacid for both lymphocyte proliferation and parasite survival [38], [39]. Hence, the expression of IDO in leishmaniasis lesions may regulate the immune response and aid in parasite eradication. Its expression is induced by inflammatory cytokines, including IFN-γ [40], [41], and by Tregs through interaction of CTLA-4 and B7 molecules [42]. Therefore, *IDO* transcription in the skin may reflect both the inflammatory environment and Treg function. In this study, we found that the expression of *IDO* was correlated with that of *IFNG* and not to *FOXP3* in CDL patients. This indicates that IDO induction in APCs is not a likely mechanism of suppression used by Tregs that infiltrate sites of ongoing immune responses to *Leishmania* in CDL patients. Rather, *IDO* expression reflects the reduced inflammatory environment present at these sites, which is consistent with increased function of a subset of Tregs after treatment.

The relatively small sample size limited our ability to detect statistically significant differences in the expression of the genes *IFNG* and *IDO* between AI (healing/clinical resistance) and patients with chronic disease (non-healing/clinical susceptibility) even though several of the latter showed marked upregulation. Furthermore, although we did not determine the mechanism of regulation by CD4^+^CD25^+^ cells, the results of functional as well as phenotypic analyses provide compelling evidence of the participation of distinct Treg subsets in both pathogenesis and resolution of CDL caused by *Leishmania* species of the *(Viannia)* subgenus. Further characterization of these subsets is warranted since immunotherapeutic strategies targeting these regulatory cells could promote healing of recalcitrant presentations of leishmaniasis.

## Supporting Information

Figure S1 **Gating strategy for analysis of CD4^+^CD25^−^ cell proliferation.** CFSE labeled CD4^+^CD25^−^ cells were cultured for 5 days with antigen presenting cells (APCs), *L. panamensis*, CD4^+^CD25^+^ cells or PHA, as indicated. **A**. Gates for CFSE labeled cells were determined in the CD4^+^ region to exclude unlabeled cells from the analysis. **B**. Regions for the proliferating populations were drawn in the CFSE labeled gate based on the peaks induced by PHA and used to calculate the proliferation index (PI) with the formula PI = Σ(% in region×2^n−1^), where n is the region number. One representative subject is shown.
(TIF)
