# Supplementary figures and images for: Regulatory T Cells in the Pathogenesis and Healing of Chronic Human Dermal Leishmaniasis Caused by Leishmania (Viannia) Species
Source: PLoS Negl Trop Dis. 2012 Apr 24;6(4):e1627. doi: 10.1371/journal.pntd.0001627 (PMC3335885; doi:10.1371/journal.pntd.0001627)

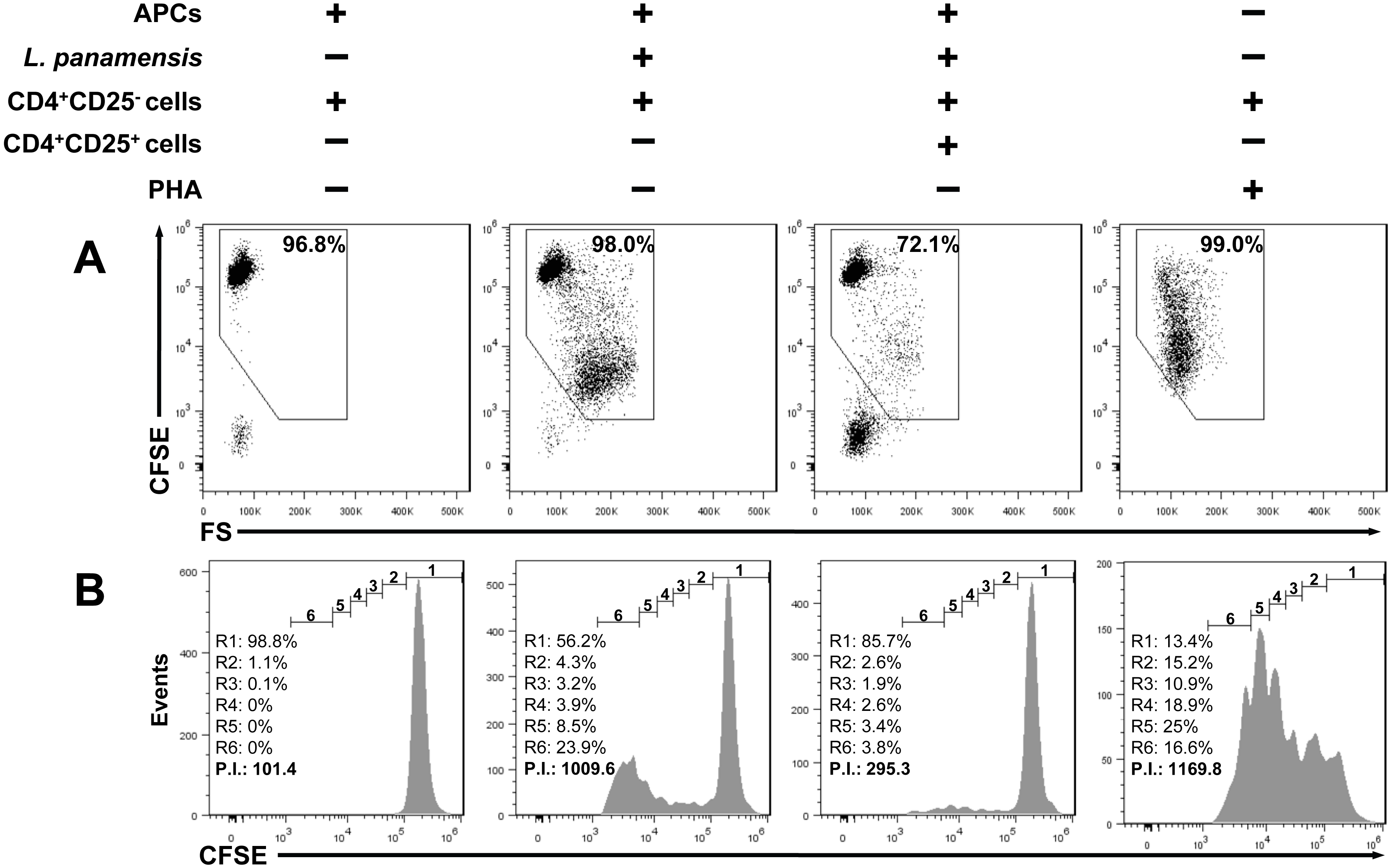

Supplement: Figure S1 — Gating strategy for analysis of CD4+CD25− cell proliferation. CFSE labeled CD4+CD25− cells were cultured for 5 days with antigen presenting cells (APCs), L. panamensis, CD4+CD25+ cells or PHA, as indicated. A. Gates for CFSE labeled cells were determined in the CD4+ region to exclude unlabeled cells from the analysis. B. Regions for the proliferating populations were drawn in the CFSE labeled gate based on the peaks induced by PHA and used to calculate the proliferation index (PI) with the formula PI = Σ(% in region×2n−1), where n is the region number. One representative subject is shown. (TIF) [file pntd.0001627.s001.tif]
